# Leveraging the science of stress to promote resilience and optimize mental health interventions during adolescence

**DOI:** 10.1038/s41467-022-33416-4

**Published:** 2022-09-28

**Authors:** Dylan G. Gee, Lucinda M. Sisk, Emily M. Cohodes, Nessa V. Bryce

**Affiliations:** 1 Department of Psychology, Yale University, New Haven, CT 06511 USA; 2 Department of Psychology, Harvard University, Cambridge, MA 02138 USA

**Keywords:** Human behaviour, Stress and resilience

## Abstract

Adolescence is marked by heightened stress exposure and psychopathology, but also vast potential for opportunity. We highlight how researchers can leverage both developmental and individual differences in stress responding and corticolimbic circuitry to optimize interventions during this unique developmental period.

## Stress and mental health in adolescence

Stress is a potent risk factor for psychopathology that is salient during adolescence. Stressful life events increase considerably in adolescence, and cross-species evidence suggests that the brain may be particularly sensitive to the negative effects of stress during this period^1^. Indeed, adolescence is characterized by heightened vulnerability: the majority of mental health disorders emerge during this stage of development, with adolescents exposed to stress earlier in life at elevated risk^2^. At the same time, adolescence is a period of immense opportunity, as heightened plasticity and the state of the developing brain confer unique strengths for coping with stress^3^.

**Fig. 1:**
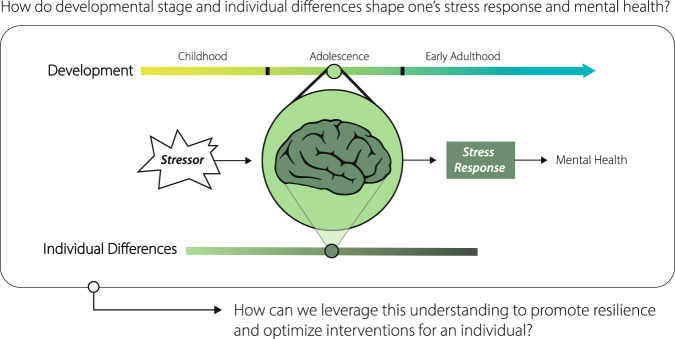
Framework for an approach to research that considers developmental and individual differences in stress responding to inform optimization of mental health interventions. Cross-species evidence has demonstrated heterogeneity in mental health following stress exposure. Understanding how an individual responds to a stressor can inform how best to promote resilience or intervene to reduce stress-related psychopathology. Here we conceptualize the stress response as multifaceted, encompassing changes in neurobiological and endocrine function, subjective experiences, and thoughts, feelings, and behaviors. Differences across development and across individuals can explain variation in responses to stress and mental health. Given dynamic changes in neurobiological systems governing stress responding across development, adolescents, on average, exhibit stress responses and mental health outcomes that differ from children and adults. Many factors that vary across individuals, such as predisposing genetic and biological factors, and variability in life experiences and the current environment, will contribute to differences in stress responding and mental health. Variability in a given factor that differs across individuals is depicted via a spectrum of shading. Together, developmental timing and individual variability will contribute to how a given individual responds to a stressor, to their mental health, and, ultimately, to how interventions could be tailored to be most effective for a given individual with stress-related psychopathology.

Understanding how people respond to stress is critical for identifying targets for intervention. In particular, delineating how stress responding differs across development and across individuals can inform whom may benefit from specific interventions and how to optimize interventions for specific developmental stages or profiles of stress exposure^4^. As scientists and mental health professionals alike grapple with the mental health crisis among youth–including a high burden of psychopathology, limited access to care, and large-scale societal stressors^5^, we offer a framework for how research can leverage the science of stress and adolescent brain development to promote resilience (Fig. 1). Here we define resilience as favorable mental health outcomes despite exposure to stress and conceptualize the processes contributing to these outcomes as dynamic and occurring across multiple systems and levels within the broader social context^6^.

## Fostering resilience by targeting the adolescent brain

While evidence-based interventions for adolescents with stress-related disorders can be highly effective, there is immense need to enhance prevention and to optimize interventions for the many youth who do not benefit sufficiently from current treatments^7^. Up to 50% of individuals at all ages do not respond sufficiently to exposure-based therapies for anxiety disorders and posttraumatic stress disorder, with evidence for similar efficacy across children, adolescents, and adults^8^. However, the factors contributing to insufficient response rates, and thus optimal approaches to enhance treatment efficacy, may differ by age group^9^. Delineating how mechanisms of fear reduction and stress coping vary across development can inform efforts to optimize interventions based on the developing brain. Indeed, adolescence is marked by dynamic changes in stress reactivity and the neurobiological systems governing stress responding, including the hypothalamic-pituitary-adrenal axis and corticolimbic circuitry^1^. Thus, optimal strategies for adaptive coping with stress and interventions most likely to promote resilience are likely to differ for adolescents relative to children or adults^7^.

Exposure-based therapies are based upon principles of fear extinction, which relies on connections between the ventromedial prefrontal cortex (vmPFC) and amygdala. Cross-species evidence has shown diminished fear extinction during adolescence^10^, corresponding to a time of protracted development of regulatory connections between the vmPFC and amygdala^11^. Stress alters these same connections, and early-life stress may lead to a shift in frontoamygdala development that could predate the onset of anxiety disorders and constrain flexibility for coping with fear and stress^12^. These findings suggest that adolescents with stress-related psychopathology may benefit from efforts to optimize fear reduction through mechanisms that target alternative neural circuitry^7^, for example by bypassing prefrontally-mediated pathways (e.g., see “Reducing Fear via Safety Signal Learning”) or by targeting connections that are relatively stronger during adolescence (e.g., see “Promoting Active Coping via Stressor Controllability”). Even beyond efforts to promote resilience following stress during adolescence, harnessing insights about the adolescent brain could leverage the plasticity of this period to potentially reshape neural systems that were disrupted by stress earlier in development^3^.

## Parsing heterogeneity across individuals to elucidate pathways of resilience

Complementing approaches that target developmental differences in stress responding, delineating individual-level factors that relate to neurodevelopment and mental health following stress–such as profiles of stress exposure–is critical for optimizing interventions^13^. Adolescents who experienced stress earlier in life are generally at higher risk for psychopathology, but there is vast heterogeneity in early-life stress exposure and in neurobehavioral phenotypes following early-life stress. Parsing such heterogeneity—in the nature, timing, and experiential elements of stress exposure, as well as in developmental trajectories following stress—can advance insights into the mechanisms linking stress with mental health^14, 15^. Alongside more traditional approaches that have been used to test predictions about specific elements or timing of stress, data-driven computational approaches can facilitate empirical derivation of key features of exposure, identify developmental windows of risk, and identify subgroups of adolescents with more uniform trajectories^4^. For example, a recent study applied similarity network fusion to large-scale environmental and neuroimaging data to decompose heterogeneous associations between brain structure and specific experiences during development^16^. Identifying subgroups of youth with more homogenous brain-environment associations enhanced prediction of mental health symptoms, suggesting that parsing individual differences in associations between the early environment and neurodevelopment may enhance identification of trajectories associated with risk versus resilience.

## Considering intersections between developmental and individual differences

**Fig. 2:**
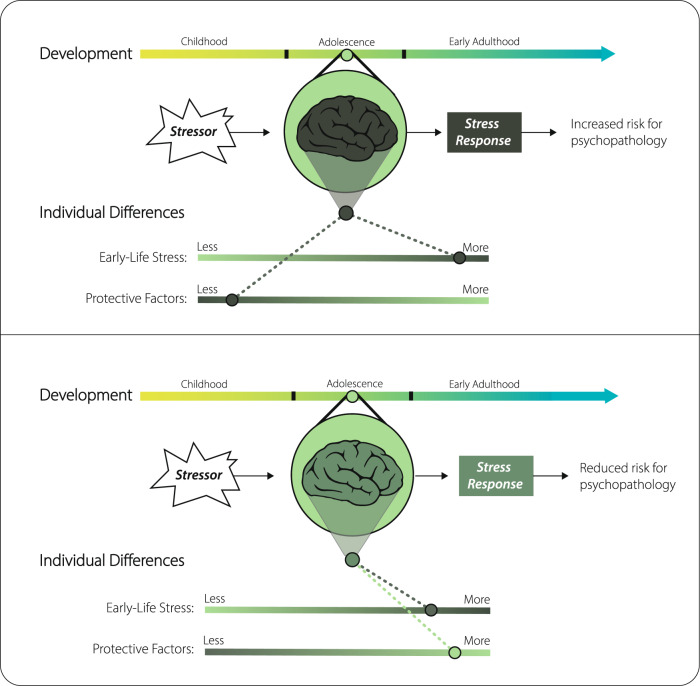
Developmental stage and individual profile of early-life stress exposure and protective factors shape stress response and mental health in the context of a stressor. The general framework of developmental and individual differences in stress responding can be applied flexibly to many specifics of developmental timing and individual factors. Here we illustrate one example of variability in exposure to early-life stress and protective factors (e.g., supportive caregiving). For a given adolescent, developmental stage, early-life stress history, and protective factors contribute meaningful information about a probable response to a current stressor. On average, an adolescent with substantial early-life stress exposure and fewer protective factors will be at higher risk for psychopathology (top panel) than an adolescent with similarly high early-life stress exposure but more protective factors (bottom panel).

Research that carefully considers both developmental and individual differences, and their interactions, will provide even richer empirical knowledge to guide tailored interventions (Fig. 2). Identifying sensitive periods and delineating developmental patterns of experience-driven plasticity are critical for understanding the onset of stress-related psychopathology and how to optimize interventions. Individual differences in stress exposure can be leveraged to better understand mechanisms of experience-driven plasticity^17^. Heightened plasticity during adolescence may amplify individual differences in neurodevelopment or mental health that emerge following experiences of stress in both positive and negative ways^3^, and latent effects of stress exposure that occurred earlier in life may also manifest most strongly during adolescence, relative to childhood or adulthood^18–20^. Moreover, cross-species evidence demonstrates that stress exposure can alter the timing of sensitive periods themselves^3, 17^.

While sensitive periods associated with experience-expectant learning often occur earlier in development, recent evidence points to opportunities for reshaping of the hypothalamic-pituitary-adrenal axis during adolescence among individuals in supportive caregiving environments who previously experienced institutionalized care^21^ and to a potential adolescent sensitive period for social reward learning^22^. These findings may suggest that interventions focused on supportive relationships or rewarding interactions with peers could be especially effective during adolescence. Future research will be important for testing which timing-related effects of stress during development are consistent with experience-expectant versus experience-dependent learning^17^ and for further clinical translation.

Examining specific experiential elements and developmental timing of stress exposure can elucidate differences in neurodevelopment and mental health during adolescence, with the potential to inform when and for whom interventions will be most effective^3, 14^. Effectively parsing heterogeneity across development and individuals will require complementary approaches—such as experimental paradigms that can isolate specific dimensions or timing of stress exposure, as well as computational approaches that can identify patterns associated with naturalistic variation in stress exposure across the lifespan. To illustrate application of this approach, below we provide two examples of key domains in which novel insights from basic science could advance knowledge of stress responding and inform optimization of interventions for adolescents.

## Reducing fear via safety signal learning

Building upon prior research that aims to enhance fear reduction beyond traditional extinction^7^, safety signal learning (a class of conditioned inhibition) may provide a promising approach to reduce excessive fear following stress during adolescence. In safety signal learning, a cue that is overly trained to signal the absence of threat is used to reduce fear in the face of a threatening cue. In contrast to extinction, where a previously threatening cue is presented repeatedly without the aversive outcome, this approach involves associating distinct environmental stimuli (i.e., safety signals) with the non-occurrence of aversive events^23^. While the neural mechanisms supporting safety signal learning continue to be explored, particularly during development, growing cross-species evidence suggests that this approach does not rely primarily on vmPFC-amygdala connections and instead involves a pathway between the hippocampus and dorsal anterior cingulate cortex (prelimbic cortex in rodents)^24^. Given evidence of protracted vmPFC-amygdala development and augmented hippocampal-prelimbic cortex connectivity during the adolescent period in rodents^25^, judicious application of safety signals to enhance fear reduction could be particularly useful during adolescence^9^. A variety of biological and environmental factors–such as current and prior exposure to trauma–are likely to contribute to individual differences in the extent to which adolescents benefit from safety learned via conditioned inhibition. Importantly, whereas stress disrupts extinction learning, recent evidence in rodents suggests that safety signals may be a robust approach to fear reduction even following stress—rodents exposed to prior stress showed impaired fear extinction, but no disruption in conditioned inhibition^26^. Moreover, evidence in rodents suggests that adolescence may be a unique period when conditioned inhibition is robust to effects of stress experienced in childhood^27^. These findings suggest that safety signal learning could target an alternative neural circuit to promote resilience beyond traditional extinction-based approaches during adolescence.

## Promoting active coping via stressor controllability

Closer examination of the conditions in which resilience is prominent can provide clues for novel intervention targets. In contrast to traditional conceptualizations of stress as universally negative, stress that is controllable has been associated with more favorable outcomes across species. Rodent studies and studies in adult humans suggest that the experience of controllable stress (versus uncontrollable stress, or no stress at all) may buffer an individual against negative effects of that stressor, as well as subsequent stress exposure^28^. Stressor controllability—the extent to which an individual has the “ability to alter the onset, termination, duration, intensity, or pattern of a stressor”^29^—may be particularly relevant during adolescence when individuals experience increasing independence from caregivers and engage in greater exploration of broader environments. One possibility is that controllable stress fosters a more active mode of coping via modulation of frontostriatal-amygdala circuitry^28^. The state of this circuitry during adolescence, including heightened striatal activation and stronger amygdala projections to the ventral striatum^11^, may render adolescents more amenable to adaptive effects of controllability than children or adults^14^. Future research delineating factors that modulate an individual’s ability to detect or leverage opportunities for control, such as prior experiences and perceptions of control, may inform for which adolescents and under which conditions controllable stress is most likely to confer resilience.

Given salient psychosocial characteristics of adolescence and the unique state of frontostriatal-amygdala circuitry at this time, adolescents may benefit from novel interventions that leverage opportunities for control to promote motivated action, or from optimizing existing practices in cognitive-behavioral therapies that involve behavioral activation and active coping^30^. While the effects of controllable stress on later stress responding remain to be tested during human development, exposure to controllable stress during the adolescent period in rodents mitigated the negative effects of uncontrollable stress in adulthood^31^, suggesting that systematic exposure to controllable stress during adolescence could have long-term benefits for mental health in the face of future stress. Among adolescents with a prior history of early adversity, stressors outside of one’s control have been more closely linked with psychopathology than stressors at least partially influenced by the individual^32^. However, meta-analytic evidence of limited differentiation in mental health by stressor type^33^ suggests that future research warrants examination of key moderators that may relate to effects of stressor controllability (e.g., for which adolescents, or under which circumstances, is controllability related to risk for psychopathology?). Outside of the laboratory setting, stress that is controllable is more likely to be characterized by social and interpersonal elements, which are highly salient during adolescence^34^. Thus, opportunities to leverage control or to optimize interventions for adolescents may especially benefit from targeting experiences of social stress.

## Conclusions

Adolescence is marked by heightened stress exposure, stress reactivity, and risk for psychopathology, as well as vast potential for resilience. Discoveries about the impacts of stress on the developing brain provide novel insights that can inform strategies to promote resilience and to enhance the efficacy of interventions for stress-related psychopathology during adolescence. Specifically, we propose that knowledge of developmental and individual differences in stress responding and related neural circuitry can guide efforts to target the unique state of the adolescent brain while tailoring optimization based on individual-level factors such as profiles of stress exposure. Guided by translation across species, this framework for leveraging the science of stress can promote mental health during and beyond the dynamic period of adolescence.
